# Osteoclast microRNA Profiling in Rheumatoid Arthritis to Capture the Erosive Factor

**DOI:** 10.1002/jbm4.10776

**Published:** 2023-06-05

**Authors:** Nguyen Hoang Dong, Lortie Audrey, Mbous Nguimbus Leopold, Marrugo Javier, Allard‐Chamard Hugues, Bouchard Luigi, Boire Gilles, Michelle S Scott, Roux Sophie

**Affiliations:** ^1^ Department of Biochemistry and Functional Genomics University of Sherbrooke and Research Centre of the Centre Intégré Universitaire de Santé et Services Sociaux de l'Estrie – Centre Hospitalier Universitaire de Sherbrooke (CIUSSSE‐CHUS) Sherbrooke Canada; ^2^ Division of Rheumatology, Department of Medicine, Faculty of Medicine and Health Sciences University of Sherbrooke and Research Centre of the Centre Intégré Universitaire de Santé et Services Sociaux de l'Estrie – Centre Hospitalier Universitaire de Sherbrooke (CIUSSSE‐CHUS) Sherbrooke Canada; ^3^ Department of Medical Biology CIUSS du Saguenay‐Lac‐Saint‐Jean Hôpital Universitaire de Chicoutimi Saguenay Canada

**Keywords:** MACHINE LEARNING, MICRORNAS, OSTEOCLAST, PREDICTIVE MODEL, RHEUMATOID ARTHRITIS

## Abstract

In rheumatoid arthritis (RA), only a subset of patients develop irreversible bone destruction. Our aim was to identify a microRNA (miR)‐based osteoclast‐related signature predictive of erosiveness in RA. Seventy‐six adults with erosive (E) or nonerosive (NE) seropositive RA and 43 sex‐ and age‐matched healthy controls were recruited. Twenty‐five miRs from peripheral blood mononuclear cell (PBMC)‐derived osteoclasts selected from RNA‐Seq (discovery cohort) were assessed by qPCR (replication cohort), as were 33 target genes (direct targets or associated with regulated pathways). The top five miRs found differentially expressed in RA osteoclasts were either decreased (hsa‐miR‐34a‐3p, 365b‐3p, 374a‐3p, and 511‐3p [E versus NE]) or increased (hsa‐miR‐193b‐3p [E versus controls]). In vitro, inhibition of miR‐34a‐3p had an impact on osteoclast bone resorption. An integrative network analysis of miRs and their targets highlighted correlations between mRNA and miR expression, both negative (*CD38*, *CD80*, *SIRT1*) and positive (*MITF*), and differential gene expression between NE versus E (*GXYLT1, MITF*) or versus controls (*CD38, KLF4*). Machine‐learning models were used to evaluate the value of miRs and target genes, in combination with clinical data, to predict erosion. One model, including a set of miRs (predominantly 365b‐3p) combined with rheumatoid factor titer, provided 70% accuracy (area under the curve [AUC] 0.66). Adding genes directly targeted or belonging to related pathways improved the predictive power of the model for the erosive phenotype (78% accuracy, AUC 0.85). This proof‐of‐concept study indicates that identification of RA subjects at risk of erosions may be improved by studying miR expression in PBMC‐derived osteoclasts, suggesting novel approaches toward personalized treatment. © 2023 The Authors. *JBMR Plus* published by Wiley Periodicals LLC on behalf of American Society for Bone and Mineral Research.

## Graphical abstract

In rheumatoid arthritis (RA), only a subset of patients develop irreversible bone destruction. In PBMC‐derived osteoclasts, a set of miRs was found to be differentially expressed in osteoclasts from patients with erosive versus non‐erosive RA or controls, modulating pathways such as mTOR signaling, osteoclast‐immune cell interactions, or fusion and fission processes. Our results demonstrate that the intrinsic properties of these cells could be used to model a predictive tool for the development of bone erosions in RA, although more readily available surrogates will need to be identified.

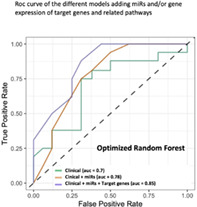

## Introduction

Epigenetic and genetic defects in noncoding RNAs are common hallmarks of human diseases.^(^ ^1^ ^)^ Noncoding RNAs are involved in the regulation of gene expression^(^ ^1^, ^2^ ^)^ and can be used as biomarkers in non‐neoplastic diseases, provided they are derived from pathogenic cells.^(^ ^3^ ^)^ Noncoding RNAs include microRNAs (miRs), a family of small (21–25‐nucleotides) RNAs that mainly repress gene translation, although some miRs can also upregulate gene expression under specific conditions.^(^ ^1^ ^)^

Within the single diagnostic entity of rheumatoid arthritis (RA), bone erosions occur in only a subset of patients but are still clinically relevant despite recent advances in therapies. In a current Canadian multicenter study of early RA, bone erosions were already present at presentation in 25% to 30% and new erosive disease developed over the next year in 10% to 25% more.^(^ ^4^ ^)^ No existing set of biomarkers in patients with early arthritis adequately identifies the potential to develop erosions, the elusive “erosive factor.” Adding a high serum level of 14‐3‐3η to antibodies (rheumatoid factor [RF] and anti‐citrullinated protein antibodies [ACPA]) and C‐reactive protein (CRP) somewhat improved the prediction of impending rapid erosive progression,^(^ ^5^ ^)^ but models still lack high predictive value that would be clinically actionable.^(^ ^6^ ^)^

As a multifactorial and polygenic disorder, RA involves both genetics and environment in its pathogenesis.^(^ ^7^ ^)^ Our hypothesis is that the erosive factor is a specific individual trait resulting from genetic and environmental exposures that “prime” osteoclast precursors. The resulting endophenotype of osteoclasts, ie, their intrinsic profile specific to each individual regardless of the environment in which osteoclasts are analyzed, determines or modulates these cells' aggressiveness under stress conditions, such as inflammation in arthritis. Predisposed osteoclast precursors, recruited from blood monocytes within synovitis,^(^ ^8^ ^)^ will be overactivated at the invasion front of the pannus. A miR‐based RA biomarker of erosiveness should ideally be assessed in osteoclasts, as these cells are the only effectors of bone resorption.^(^ ^3^ ^)^ Being poorly accessible in human bones, osteoclasts need to be studied after differentiation from their precursors present in peripheral blood mononuclear cells (PBMC) in long‐term culture in the presence of macrophage colony‐stimulating factor (M‐CSF) and receptor activator of NF‐κB ligand (RANKL).^(^ ^9^, ^10^ ^)^ Importantly, the phenotype of PBMC‐derived osteoclasts in vitro can be correlated with their in vivo profile.^(^ ^11^ ^)^ Although PBMC‐derived osteoclasts from RA patients exhibited higher bone resorption at baseline, ongoing treatments or the degree of RA inflammation/disease activity may affect the number of osteoclast precursors in PBMCs,^(^ ^12^ ^)^ as well as the number of osteoclasts formed in vitro, the proportion of CD14^+^ cells in PBMCs, or the raw resorption.^(^ ^10^ ^)^ On the contrary, in RA osteoclast cultures assessed repeatedly over time, some parameters such as bone resorption capacity (resorption per osteoclast) and apoptosis rate remained constant, regardless of disease activity and treatments, reflecting an intrinsic osteoclast profile that remains stable over time.^(^ ^10^ ^)^

Our first objective was to identify miR expression profiles (osteoclast‐related signature) in long‐term cultures of osteoclast precursors from patients with erosive (E) or nonerosive (NE) RA and controls. We then compared these miR expression profiles to assess their correlation with destructive outcomes.

## Patients and Methods

### Cohort and phenotype classification

RA patients positive for RF and ACPA (anti‐CCP2) were selected from a long‐term cohort of adult patients included at the onset of arthritis (“Early Undifferentiated PolyArthritis” [EUPA] cohort).^(^ ^5^ ^)^ Additional baseline clinical and biological variables included demographics, number of tender/swollen joints, complete blood count, serum CRP, and medications. Hand and feet X‐rays were obtained as per protocol and scored according to the validated Sharp/van der Heijde score (SHS; erosion score threshold for “erosive” ≥5).^(^ ^13^ ^)^ We recruited a total of 70 RA patients either clearly erosive (36 early RA [duration ≤18 months] and SHS ≥5) or nonerosive (34 with long‐term observation [≥3 years] and SHS ≤1), and 43 sex‐ and age‐matched healthy controls.

### Materials for in vitro cultures

Opti Eagle's minimum essential medium (Opti‐MEM), antibiotics, glutamine, and fetal bovine serum (FBS) were purchased from Wisent (Montreal, Canada); Ficoll–Paque from Amersham Biosciences (Montreal, Canada); and human recombinant (hr) M‐CSF and hrGM‐CSF from R&D Systems (Minneapolis, MN, USA). Soluble hrRANKL was produced in our laboratory. MiR inhibitors for 5 miRs hsa‐miR‐34a‐3p, −193b‐3p, −365b‐3p, −374a‐3p, and −511‐3p (miRIDIAN microRNA Hairpin Inhibitors), along with a nontargeting universal negative control based on cel‐miR‐67, were purchased from GE Healthcare Dharmacon, Inc. (Horizons Discovery, Lafayette, CO, USA).

### PBMC‐derived osteoclast cultures and RNA extraction

PBMCs were isolated from blood samples by density gradient, suspended in Opti‐MEM 2% FBS, and plated at 3 × 10^6^/mL in 8‐well chambers (Lab‐Tek, Bedford, MA, USA). Adherent cells were cultured for 3 weeks in the presence of M‐CSF and RANKL to generate multinucleated cells that express osteoclast markers and are capable of resorbing bone, as described.^(^ ^9^, ^14^, ^15^ ^)^ At the end of the culture, the cells were lysed in Qiazol (Qiagen, Hilden, Germany) and stored at −80°C. Total RNA was extracted using an miRNeasy Kit (Qiagen), which allows efficient purification of total mRNA and hsa‐miRs (hereafter referred to as miRs).

### Expression profiling of miRs by high‐throughput sequencing (discovery cohort)

PBMC‐derived osteoclast cultures were obtained as described above from 16 RA patients (8 E, 8 NE) and 11 age‐ and sex‐matched controls in a discovery cohort, and miRs were extracted from mature osteoclasts at the end of cultures for processing by high‐throughput sequencing. The *hg38.98 human genome annotation* (Gene Transfer Format file [GTF]) was downloaded from the Ensembl database (http://www.ensembl.org),^(^ ^16^ ^)^ in which we added 5p and 3p miR annotations from the mirBase database (http://www.mirbase.org/).^(^ ^17^ ^)^ RNA sequencing (RNA‐Seq) was performed on an Illumina HiSeq platform (Genome Quebec, Montreal, Canada) using 50‐nt single‐end reads, as previously described.^(^ ^18^ ^)^ Briefly, the samples were multiplexed, providing between 2.7 and 18.3 million reads per sample. The fastq files were trimmed using Trimmomatic v0.36 to remove the Illumina adapters.^(^ ^19^ ^)^ FastQC v0.11.8 was used for quality control.^(^ ^20^ ^)^ Alignments against the modified human genome annotation were performed using STAR v2.2.3a,^(^ ^21^ ^)^ and the aligned reads were quantified using CoCo software v0.2.2.^(^ ^22^ ^)^ The RNA sequencing pipeline was built using the Snakemake workflow system,^(^ ^23^ ^)^ and the parameters associated with each processing step are available in the Snakemake pipeline (*Github*: https://github.com/hoang31/miro_arthritis *)*. The CoCo package was downloaded from (*Github*: https://github.com/scottgroup/coco), and all other software used in the Snakemake pipeline has been downloaded from the Bioconda channel. The pipeline was executed on the Digital Research Alliance of Canada infrastructure (https://alliancecan.ca/en).

### miR gene expression analysis by TaqMan qPCR (replication cohort)

In an independent replication cohort consisting of 54 RA patients (26 NE and 28 E) as well as 32 sex‐ and age‐matched controls, RNA was extracted after osteoclast culture as described above. Based on their differential expression between the E, NE, and control groups, 25 miRs were selected from the RNA‐Seq analysis and evaluated for their expression using miR stem‐loop RT specific primers and the validated TaqMan qPCR assay, routinely designed and validated (RNomics Platform, University of Sherbrooke) (Supplemental Table S1).^(^ ^24^ ^)^ The expression levels of the target mRNAs were normalized to that of small nuclear RNA U6 (*snU6*) as an internal control.^(^ ^25^, ^26^ ^)^

### Gene expression study by qPCR from total RNA

We investigated the relative expression levels of 33 mRNAs (predicted target genes or genes associated with pathways involving the candidate miRs). All primers were designed based on sequences reported in the Aceview database (Supplemental Table S2).^(^ ^27^ ^)^ At least 50 μg of total RNA were harvested from each cell sample and processed for qPCR. Amplification and detection of the candidate mRNAs and of three reference housekeeping genes (Mitochondrial Ribosomal Protein L19 [*MRPL19*], Tyrosine 3‐Monooxygenase/Tryptophan 5‐Monooxygenase Activation Protein Zeta [*YWHAZ*], and Pumilio homolog 1 [*PUM1*]) were conducted with a Realplex 2 Master Cycler (Eppendorf, Mississauga, Canada).

### Modulation of miR expression and its impact on cultured osteoclasts in vitro

Human osteoclasts were generated in in vitro cultures from cord blood monocytes (CBMs) as a source of osteoclast precursors. Specific knockdown of the selected miRs (using anti‐miR oligonucleotides or antagomirs) or control miRs was performed by a 48‐hour transfection of osteoclast cultures at day 14 using Lipofectamine LTX (Life Technologies, Carlsbad, CA, USA), as previously described.^(^ ^15^ ^)^ On day 21 of culture, cells were fixed with 3% PFA, stained with hematoxylin and eosin, and visualized (Zeiss [Jena, Germany] ApoTome 2 microscope).

To assess the bone resorption capacity of mature osteoclasts, osteoclast precursors were initially cultured on bovine bone slices and transfected as described above. At the end of cultures, the bone slices were removed, washed with sodium hydroxide and distilled water, sonicated to remove cell debris, and stained with 1% toluidine blue containing 1% sodium borate. By light microscopy with epi‐illumination, the resorption pits appeared bright and purple, and bone resorption areas were quantified (ImageJ).^(^ ^15^, ^28^ ^)^

### Interaction network analysis

The miR‐target interactions were extracted from the miRTarBase database (mirtarbase.cuhk.edu.cn) containing experimentally validated miR‐mRNA interactions.^(^ ^29^ ^)^ The interaction networks between the selected miRs and their targets were created and visualized using Cytoscape software.^(^ ^30^ ^)^ We used the “clusterProfiler” R package for the Gene Ontology (GO) and Kyoto Encyclopedia of Genes and Genomes (KEGG) pathway enrichment analysis of candidate targets of the miRs, as previously described.^(^ ^18^ ^)^

### Machine learning

Analyses using machine‐learning models were performed to investigate the potential contribution of clinical variables and qPCR expression levels of differentially expressed miRs and of target genes to classify patients with erosive versus nonerosive RA. For this aim, clinical data (Supplemental Table S3) were formatted and encoded using the scikit‐learn python library (v0.23.2). We completed the missing values using a K‐nearest neighbors (KNN) imputation method (KNNImputer from the scikit‐learn python library). The resulting data were then split into hyperparameter tuning (20%) and training (80%) sets. The optimal hyperparameters of three different models (logistic regression, KNN, and random forest) were identified using the grid search function (available from the scikit‐learn library) on the tuning set. We then assessed model performance of the classification (accuracy, error rate, specificity, sensitivity, area under the curve [AUC]) by performing leave‐one‐out cross‐validation (LOOCV) on the training set. Variable contributions for each independent model were measured by calculating the Shapley additive explanations (SHAP) values^(^ ^31^ ^)^ with the SHAP python library (v0.39.0). To optimize the accuracy and AUC of the models, feature selection was performed using the Boruta‐Shap python library (v1.0.16) algorithm using LOOCV of the training set. All selected features considered important for the classification were merged and then used to train new models.

### Statistical analysis

Subject demographics were compared among groups using chi‐square and Wilcoxon signed‐rank tests, as appropriate. We used paired ANOVA to compare in vitro conditions and paired *t* tests for antagomir validation (PRISM software 8.0). *RNA‐Seq*: Differential gene expression analysis using R's “DESeq2” package (v1.26.0) was performed to compare RNA‐Seq results among groups.^(^ ^32^ ^)^ The raw counts quantified by CoCo were input into DESeq2 for the gene expression analysis. Log2 fold change values and *p* values corrected by false discovery rate (FDR) were calculated. From these results, we selected the top 25 miR candidates for qPCR analysis based on the following thresholds: either *p* value <0.01 and FDR 0.35 or *p* value <0.05 together with a fold‐change >2. *Gene expression of miRs and target mRNAs*: We used Wilcoxon signed‐rank test to compare the expression of the tested miRs among groups. The calculated *p* values were then adjusted using FDR correction. Statistical significance was defined as an adjusted value of *p* < 0.05. In a previous study profiling miRs in pagetic osteoclasts, a difference in miRs expression (Taqman qPCR) could be demonstrated by comparing two age‐ and sex‐matched groups of 21 subjects (α 0.5);^(^ ^18^ ^)^ we assumed similar power considerations and similar sample sizes for the present study.

## Results

### Cohort description and study design

**Fig. 1:**
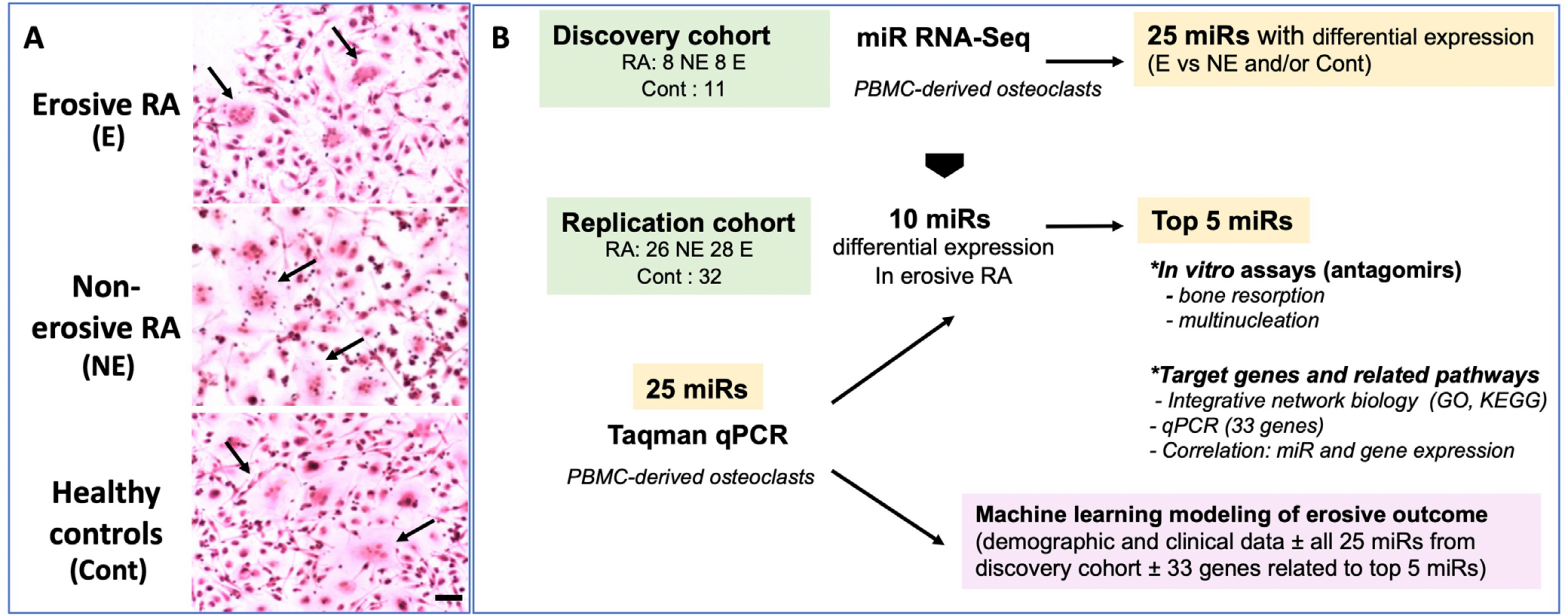
Multistep evaluation of microRNAs (miRs) as elements of erosiveness in rheumatoid arthritis (RA). We first investigated miRs by RNA‐Seq in in vitro peripheral blood mononuclear cell (PBMC)‐derived osteoclasts from patients with erosive (E) or nonerosive (NE) RA and age‐ and sex‐matched healthy controls in a small discovery cohort. (*A*) Osteoclast cultures. Illustration of PBMC‐derived osteoclast cultures (arrows) (scale bar = 10 μm). (*B*) Study design. Expression of 25 differentially expressed miRs was then verified by RT‐qPCR in a larger independent replication cohort. The 5 miRs with the most significant variations in RT‐qPCR were evaluated in vitro and integrated in an interaction network to study their targets and related pathways (GO, KEGG, and gene expression in mature osteoclasts). Finally, using machine learning, we determined predictive models for the development of bone erosions in RA including both clinical data and the 25 miRs.

The discovery cohort consisted of 27 individuals, including 16 RA patients (8 E and 8 NE) with a sex ratio of 1:1 and a mean age of 61.4 ± 11 years (39–75 years) and 11 age‐ and sex‐matched controls with a mean age of 65.45 ± 11 years (36–72 years). The replication cohort (*n* = 86) described in Table 1 consisted of 54 RA patients (26 NE and 28 E), female/male ratio of 3 to 2, and a mean age of 63 ± 10 years (33–83 years), with 32 age‐ and sex‐matched controls; the cohort included 21 age‐ and sex‐matched triplets. Demographics were similar among groups (age, sex, body mass index [BMI]); among RA subjects, disease activity (SJC, TJC, CRP) was also similar in E versus NE, whereas erosive patients used biotherapies more frequently (*p* < 0.01) and less calcium and vitamin D supplements (*p* < 0.05). All RA patients were ACPA‐positive, all but one were RF‐positive, and the RF titer was higher in E versus NE RA (*p* < 0.05) (Table 1). The study consisted of the analysis of PBMC‐derived osteoclasts obtained in long‐term culture in the different groups and included a multistep evaluation of miRs as components of the erosive factor (study design in Fig. 1).

**Table 1:** Description of the Replication Cohort

	Replication cohort		
	Controls	Nonerosive RA (NE)	Erosive RA (E)
	*n* = 32	*n* = 26	*n* = 28
Sex distribution	17 M, 15 F	7 M, 19 F	14 M, 14 F
Age (years) mean (min–max)	61.6 (32–82)	61.6 (36–83)	63.6 (33–81)
BMI mean (SD)	27.7 (4.8)	26.2 (5)	27.6 (5.7)
Menopause % F	12/15	16/19	13/14
Smoking	12 no, 2 active, 15 past	11 no, 3 active, 10 past	8 no, 8 active, 11 past
Alcohol	7 no, 18 active, 3 past	5 no, 12 active, 8 past	4 no, 10 active, 12 past
Serum 25‐OH vitamin D nmol/L mean (SD)	87 (27)	91 (26)	83 (29)
Joint stiffness (%)	n/a	56%	71%
SJC66 mean (SD)	n/a	1.8 (2.8)	1.7 (2.9)
TJC68 mean (SD)	n/a	1.8 (2.7)	1.4 (2.1)
RF (IU/mL) mean (SD)	n/a	254 (280)	428 (438)*
Anti‐CCP (U/mL) mean (SD)	n/a	181 (117)	193 (120)
CRP (mg/L) mean (SD)	3.7 (1.6)	5 (5.7)	8.4 (9.4)
Methotrexate use	n/a	88%	75%
Methotrexate (dose)	n/a	20.5 ±5	20.3 ±5
Biologics use	n/a	15%	57% **
Prednisone use	n/a	8%	11%
Osteoporosis treatment	n/a	12% (2 BP, 1 denosumab)	21% (BP)
Calcium‐vitamin D use	n/a	85%	57%*

Abbreviation: BMI = body mass index; BP = bisphosphonate; CRP = C‐reactive protein; RF = rheumatoid factor.

* *p* < 0.05.

** *p* < 0.01 (E versus NE).

### DeepSeq of osteoclast miRs (discovery cohort) and qPCR expression (replication cohort) identify 5 miRs of interest differentially expressed between osteoclasts from erosive vs nonerosive RA

Using patients from the discovery cohort, genomewide miR expression was assessed using RNA‐Seq to study the miR expression profiles in osteoclasts. The RNA‐Seq approach is far more comprehensive than RT‐qPCR to discover differentially expressed miRs.^(^ ^33^ ^)^ However, RNA‐Seq may be less sensitive and may not detect some miRs of interest. Combining the RNA‐Seq and RT‐qPCR approaches may be preferable. The “discovery” step allowed us to identify 25 miRs potentially differentially expressed among groups (E, NE, controls) (Table 2). The expression of selected miRs was then validated by qPCR and normalized to snU6 in a larger independent replication cohort (Table 1).

**Table 2:** RNA‐Seq Results (25 miRs Selected)

	miR name	log2 fold change	*p* value	*p* adj (FDR)
E versus Cont	hsa‐mir‐374a‐5p	−1.287	0.001	0.35
	hsa‐mir‐454‐3p	−1.091	0.001	0.35
	hsa‐mir‐7706	−1.029	0.002	0.35
	hsa‐mir‐374a‐3p	−0.654	0.002	0.35
	hsa‐mir‐25‐3p	−0.442	0.003	0.35
	hsa‐mir‐15b‐3p	−0.968	0.003	0.35
	hsa‐mir‐511‐3p	−1.296	0.003	0.35
	hsa‐mir‐15b‐5p	−1.123	0.004	0.35
	hsa‐mir‐142‐5p	−0.888	0.005	0.35
	hsa‐mir‐7‐1‐3p	−1.269	0.005	0.35
	hsa‐mir‐34a‐3p	−1.654	0.005	0.35
	hsa‐mir‐23b‐3p	−0.802	0.005	0.35
	hsa‐mir‐17‐5p	−1.115	0.006	0.35
	hsa‐mir‐200c‐3p	−0.715	0.006	0.35
	hsa‐mir‐374c‐5p	−1.425	0.007	0.35
	hsa‐mir‐1246	−1.431	0.007	0.35
	hsa‐mir‐147b‐5p	−0.908	0.007	0.35
	hsa‐mir‐194‐2‐5p	−1.066	0.008	0.35
	hsa‐mir‐193b‐3p	1.544	0.011	0.37
	hsa‐mir‐365a‐3p	0.60	0.091	0.62
NE versus Cont	hsa‐mir‐152‐3p	−0.534	0.000	0.06
	hsa‐mir‐365b‐3p	−4.131	0.009	0.79
	hsa‐mir‐1‐1‐3p	3.008	0.033	0.93
E versus NE	hsa‐mir‐193b‐3p	−2.117	0.001	0.67
	hsa‐mir‐106a‐5p	−2.120	0.008	0.81
	hsa‐mir‐365b‐3p	4.734	0.009	0.81
	hsa‐mir‐29b‐1‐3p	−2.783	0.034	0.95
	hsa‐mir‐206	−2.230	0.036	0.95

Abbreviation: Cont = controls; FDR = false discovery rate; E = erosive rheumatoid arthritis; miR = microRNA; NE = nonerosive rheumatoid arthritis.

**Fig. 2:**
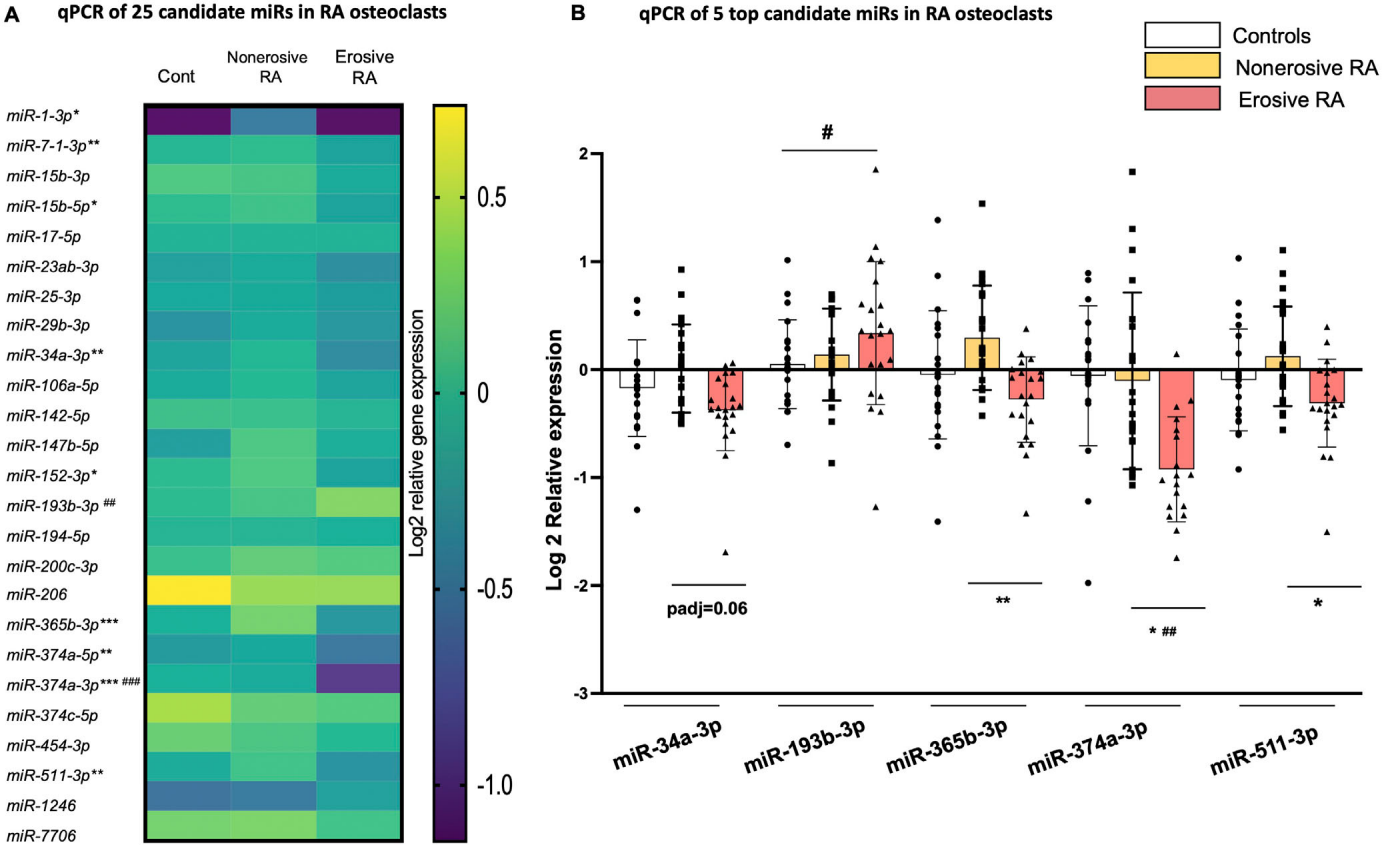
MicroRNA (miR) expression by RT‐qPCR in rheumatoid arthritis (RA) osteoclasts. miR RNA extraction was performed at the end of osteoclast cultures, followed by real‐time RT‐qPCR of the 25 selected miRs. miR expression was normalized to snU6 in peripheral blood mononuclear cell (PBMC)‐derived osteoclasts in patients with established erosive (E) or nonerosive (NE) RA and in age‐ and sex‐matched controls (Cont), *n* = 21/group. (*A*) Expression of 25 miRs in osteoclast cultures. The results are presented as a heat map reflecting the log2 relative expression in the 3 groups: controls, nonerosive RA, and erosive RA. The *p* values are indicated: *p < 0.05, **p < 0.01, ***p < 0.001 (E versus NE), ^##^ *p* < 0.01, ^###^ *p* < 0.001 (Cont versus E). (*B*) Five top miRs differentially expressed in osteoclast cultures. Boxplot: mean, SD; the FDR adjusted *p* values are indicated: *p adj <0.05, **p adj <0.01 (E versus NE), ^#^ *p* adj <0.05, ^##^ *p* < 0.01 (Cont versus E).

Using qPCR, 10 mature miRs were found to be differentially expressed in osteoclasts from erosive RA (Fig. 2*A*). After FDR correction, the top five miRs were miR‐365b‐3p, 374a‐3p, and 511‐3p, whose expression remained significantly decreased in E osteoclasts compared with NE osteoclasts, with a trend toward decrease for miR‐34a‐3p, whereas miR‐193b‐3p expression remained significantly increased (Fig. 2*B*).

The difference in expression between E and NE assessed by qPCR and RNA‐Seq did not match for all candidate miRs. For example, miR‐365b‐3p expression was very low or even undetectable in several samples in RNA‐Seq, yielding large increases (log2 4 E versus NE) by RNA‐Seq, but a decrease was found by qPCR. This highlights the difficulty of RNA‐Seq to identify differences in expression in lowly expressed transcripts, supporting a qPCR step.^(^ ^34^ ^)^ Furthermore, most of the variation in RNA‐Seq likely results from true interindividual variations and a much smaller sample size in the discovery than in the confirmation cohort.

### Impact of miR inhibition on osteoclast multinucleation and bone resorption

**Fig. 3:**
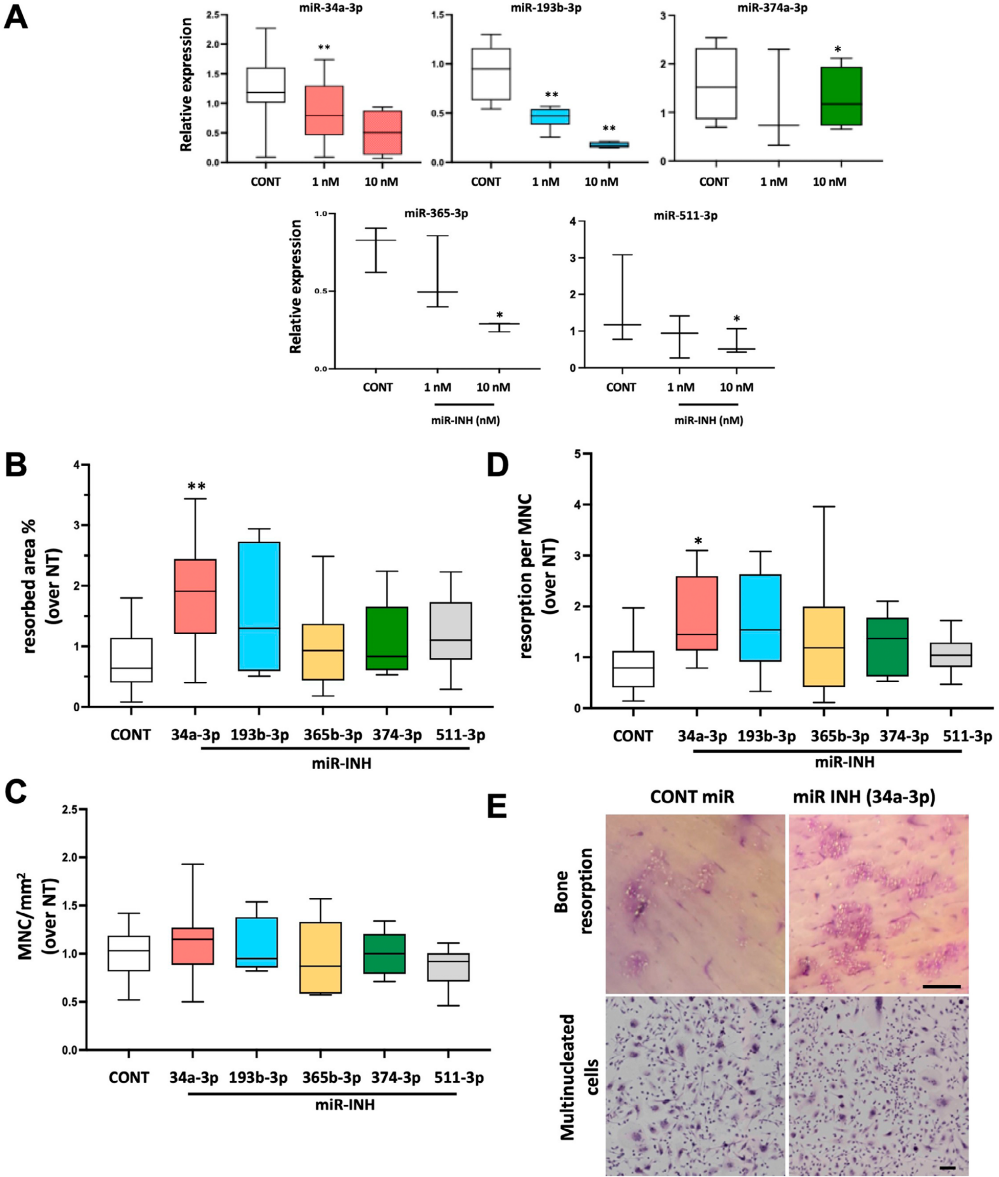
Impact of microRNA (miR) inhibition on osteoclasts. Cord blood monocyte (CBM)‐derived osteoclast cultures were either nontransfected (NT) or transfected with antagomirs (1 or 10 nM) (miR‐INH) or with a negative control (Cont) on day 14 for 48 hours; miR expression is represented graphically. (*A*) RT‐qPCR after miRNA transfection to validate antagomir transfection in mature osteoclasts. Relative expression levels of miR‐34a‐3p, 193b‐3p, 365b‐3p, 374a‐3p, and 511‐3p after inhibition with an antagomir are presented (boxplot: median, min–max, *n* = 3–6). Impact of miR antagomirs on osteoclasts. Osteoclast cultures were either nontransfected (NT), transfected with miR‐INH 1 nM (miR‐34a‐3p) or 10 nM for all others on day 14 for 48 hours, or transfected with a negative control (Cont). At day 21, the number of cells containing 3 or more nuclei was counted in each condition, and bone resorption was evaluated (ImageJ software). (*B*) The results are presented as % of resorbed area on each slice, (*C*) % of multinucleated cells (MNCs) per surface area, (*D*) resorption per MNC. All results are expressed normalized over nontransfected cultures (NT) (boxplot: median, min–max; *n* = 6–10). **p < 0.01, *p < 0.05 (versus Cont). (*E*) Representative images of the effect of miR‐34a‐3p inhibition on resorption and multinucleated cells are presented (scale bar = 10 μm).

Focusing on the top 5 miRs (34a‐3p, 193b‐3p, 365b‐3p, 374a‐3p, 511‐3p), we evaluated their impact on osteoclast formation and bone resorption using cord blood monocytes (CBMs) as osteoclast precursors, an in vitro osteoclast differentiation model validated in humans.^(^ ^15^ ^)^ The objective was to evaluate the direct impact, if any, of each candidate miR on osteoclast formation and function, so far only demonstrated in murine models for miR‐34a.^(^ ^35^ ^)^ We first tested in mature osteoclasts the efficacy of specific miR inhibitors (antagomirs) to reduce miR expression by qPCR. We obtained a significant inhibitory effect of 60% to 80% of the miR expression of 4 miRs at a concentration of 1 nM (miR‐34a‐3p) and 10 nM (miR‐193b‐3p, 365b‐3p, 511‐3p) of antagomirs. In the case of miR‐374a‐3p, 10 nM of antagomir induced only a small 18% decrease in miR expression (Fig. 3*A*). After adding specific antagomirs, bone resorption was assessed, as was the number of multinucleated cells per surface area (Fig. 3*B*). Inhibition of miR‐34a‐3p, but not that of the other miRs, led to a significant increase in bone resorption (2.5‐fold), without any change in the numbers of mutinucleated cells but an increase in the resorption per osteoclast (2‐fold increase) (Fig. 3*C*). Therefore, inhibiting miR‐34a‐3p in differentiating human osteoclasts increased bone resorption in vitro, which is in line with the negative effect of miR‐34a on osteoclastic bone resorption described in mouse models.^(^ ^35^ ^)^

The decrease in expression of miR‐374a‐3p after antagomir treatment was not sufficient to fully appreciate its impact on resorption. On the other hand, the lack of direct impact of marked inhibition of the other 3 miRs (miR‐193b‐3p, 365b‐3p, 511‐3p) is not completely surprising; the effect of miRs is that of a network rather than of a single factor, with the additive roles of a set of miRs (and probably other factors) acting on different pathways leading to resorption.

### Integrative network biology analysis of differentially expressed top 5 miRs

**Fig. 4:**
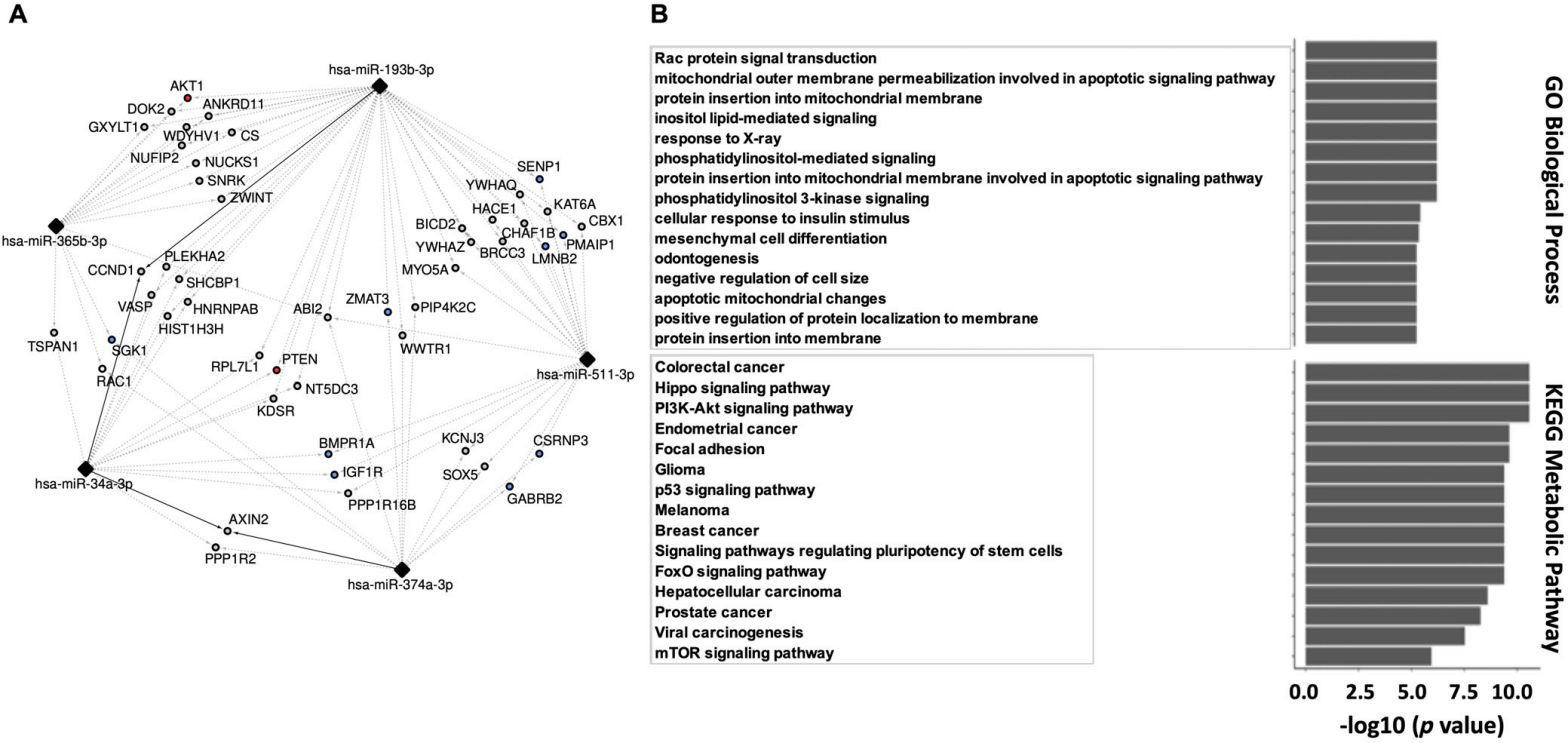
Integrative network biology analysis. (*A*) MicroRNA (miR)‐mRNA interaction network generated by Cytoscape to analyze the five candidate miRs and their target genes. The “strong” (solid line) and “weak” (dotted line) experimentally validated interactions of all five miRs (34a‐3p, 365b‐3p, 374a‐3p, 511‐3p, and 193b‐3p) were extracted. (*B*) Enrichment analysis of target genes using Gene Ontology and KEGG metabolic pathways is presented with the top 15 pathways of interest.

Next, we studied the interactions among the miRs of interest and their regulated pathways using the miRTarBase database.^(^ ^29^ ^)^ The integrative network analysis of these miRs and their targets highlighted several common pathways, often closely or remotely related to the mTOR pathway, such as *PTEN* phosphatase, kinases *AKT* ^(^ ^36^ ^)^ and *SNRK*,^(^ ^37^ ^)^ ERK signaling as *SHCBP1*,^(^ ^38^ ^)^ PI3K as *PLEKHA2*,^(^ ^39^ ^)^ and autophagy as *WDYHV1*,^(^ ^40^ ^)^ as well as potential osteoclast‐immune cell interactions such as *PLEKHA2*, related to the ICOS‐ICOSL pathway; *GXYLT1*, related to the Notch pathway;^(^ ^41^ ^)^ and *HACE1*, an E3 Ub ligase involved in innate immunity^(^ ^42^ ^)^ (Fig. 4*A*).

Direct targets of the selected 5 miRs of interest include *RAC1*, involved in membrane protrusions;^(^ ^43^ ^)^ for only miR‐193b‐3p, *SLC1A5*, encoding syncytin‐1 receptor involved in osteoclast fusion;^(^ ^11^ ^)^ and *NF1*, encoding RasGAP Neurofibromin, involved in the fission process.^(^ ^44^ ^)^

In addition, we carried out enrichment analysis of the GO and KEGG metabolic pathways. We could describe the GO terms and metabolic pathways in the group of genes interacting strongly with the five interconnected miRs. The 10 most enriched pathways included biological processes involved in apoptotic pathways and protein insertion into membrane, and metabolic pathways involved in cancers, as well as some also crucial to osteoclasts, such as PI3K‐Akt and mTOR. Finally, another pathway of interest was highlighted, as the most enriched pathway “Rac protein signal transduction,” as well as “Negative regulation of cell size,” which both include *RAC1* and involved fusion and fission processes^(^ ^43^ ^)^ (Fig. 4*B*).

### Osteoclast expression of genes targeted by the 5 candidate miRs as well as genes from related pathways as mTOR signaling and immune cell‐osteoclast interplay

**Fig. 5:**
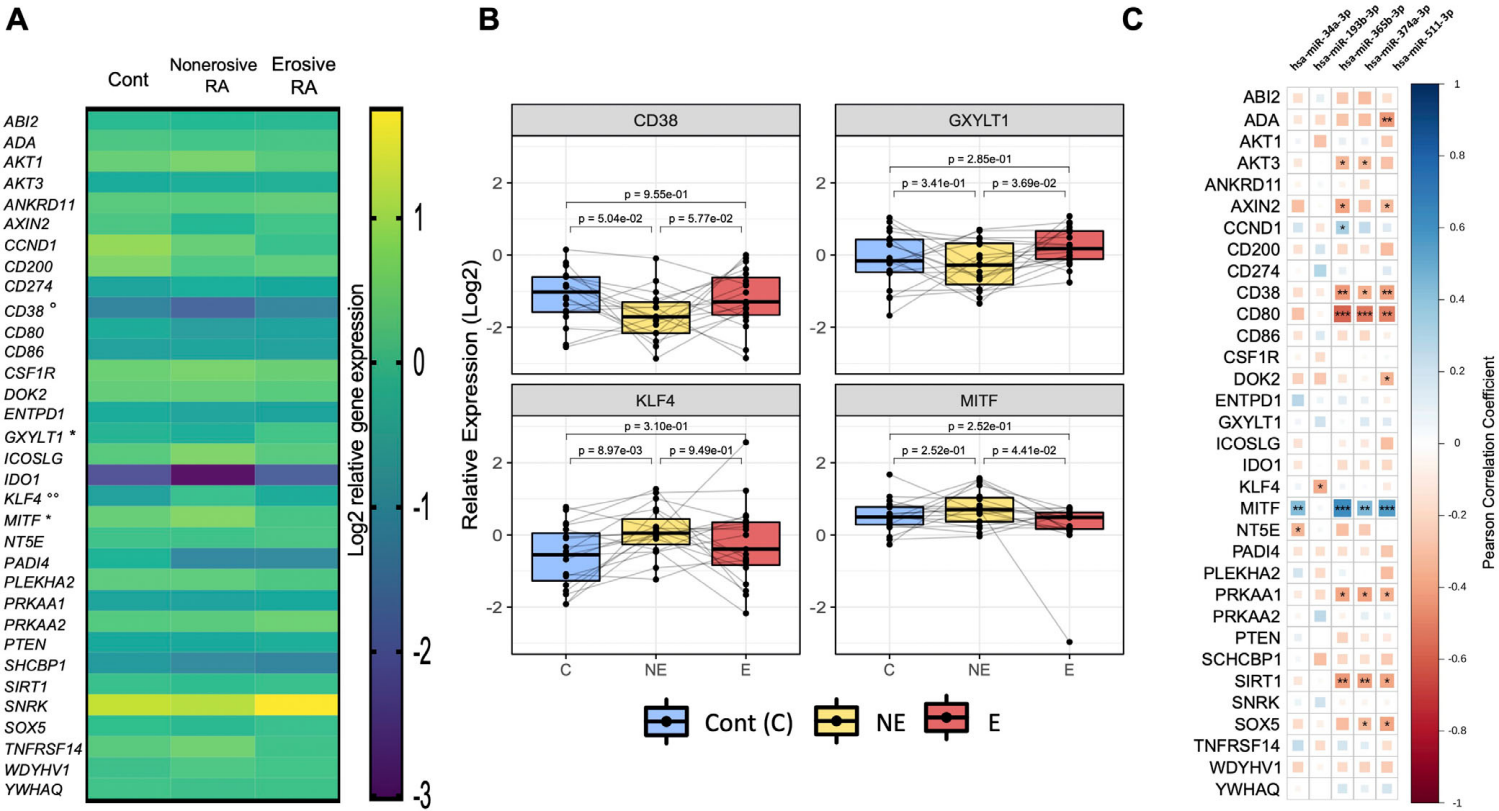
Expression of genes and pathways targeted by microRNAs (miRs) in mature osteoclasts. Whole RNA extraction was performed on mature peripheral blood mononuclear cell (PBMC)‐derived osteoclasts at the end of cultures, followed by real‐time PCR of 33 genes in established erosive (E) and nonerosive (NE) rheumatoid arthritis (RA) and age‐ and sex‐matched controls. (*A*) The results are presented as heat maps reflecting the log2 relative expression in the three groups. The *p* values are indicated: *p < 0.05 (E versus NE), °*p* < 0.05, °°*p* < 0.01 (Cont versus NE). (*B*) The four genes differentially expressed in osteoclast cultures are presented. The results are presented in log2 fold changes (paired boxplots, median, min–max; *n* = 19/group; C = controls; NE = nonerosive RA; E = erosive RA). (*C*) Correlation between miR expression and target genes and associated pathways. Correlation heatmap (positive in blue, negative in red) with significance levels expressed by asterisks (***p ≤ 0.001, **p ≤ 0.01, *p ≤ 0.05).

We selected genes concomitantly regulated by at least 2 of the 5 miRs whose expression levels were different between the groups (E, NE, controls) (14 genes), as well as some genes in related pathways of interest (mTOR signaling, immune cell‐osteoclast interplay) (19 genes) (Supplemental Table S2). The expression of these 33 genes was evaluated by qPCR in mature osteoclasts (Fig. 5*A*), revealing the differential expression of 4 genes, with a significant increase in *GXYLT1* and a decrease in *MITF* in E compared with NE, and a significant increase in *KLF4* and a decrease in *CD38* in NE compared with controls, with an increased trend in E versus NE for *CD38* (Fig. 5*B*).

To validate the biological relevance of these changes with respect to the miR variations, a correlation study was performed between the expression levels of the 5 miR candidates and those of the 33 genes. Levels of most miRs (34a‐3p, 365b‐3p, 374a‐3p, 511‐3p) were negatively correlated with the expression of *AKT3*, *AXIN2, CD38, CD80, PRKAA1, SIRT1*, and *SOX5* and positively with *MITF* (Fig. 5*C*). Correlation between the expression of the different miRs were observed; miR‐365b‐3p was strongly positively correlated with 3 of the other miRs of interest (miR‐374a‐5p [r 0.79], miR‐511‐3p [r 0.6], and miR‐34a‐3p [r 0.67]), which may suggest a common modulation of their expression.

### Machine learning to model a predictive tool for the development of bone erosions in RA

**Fig. 6:**
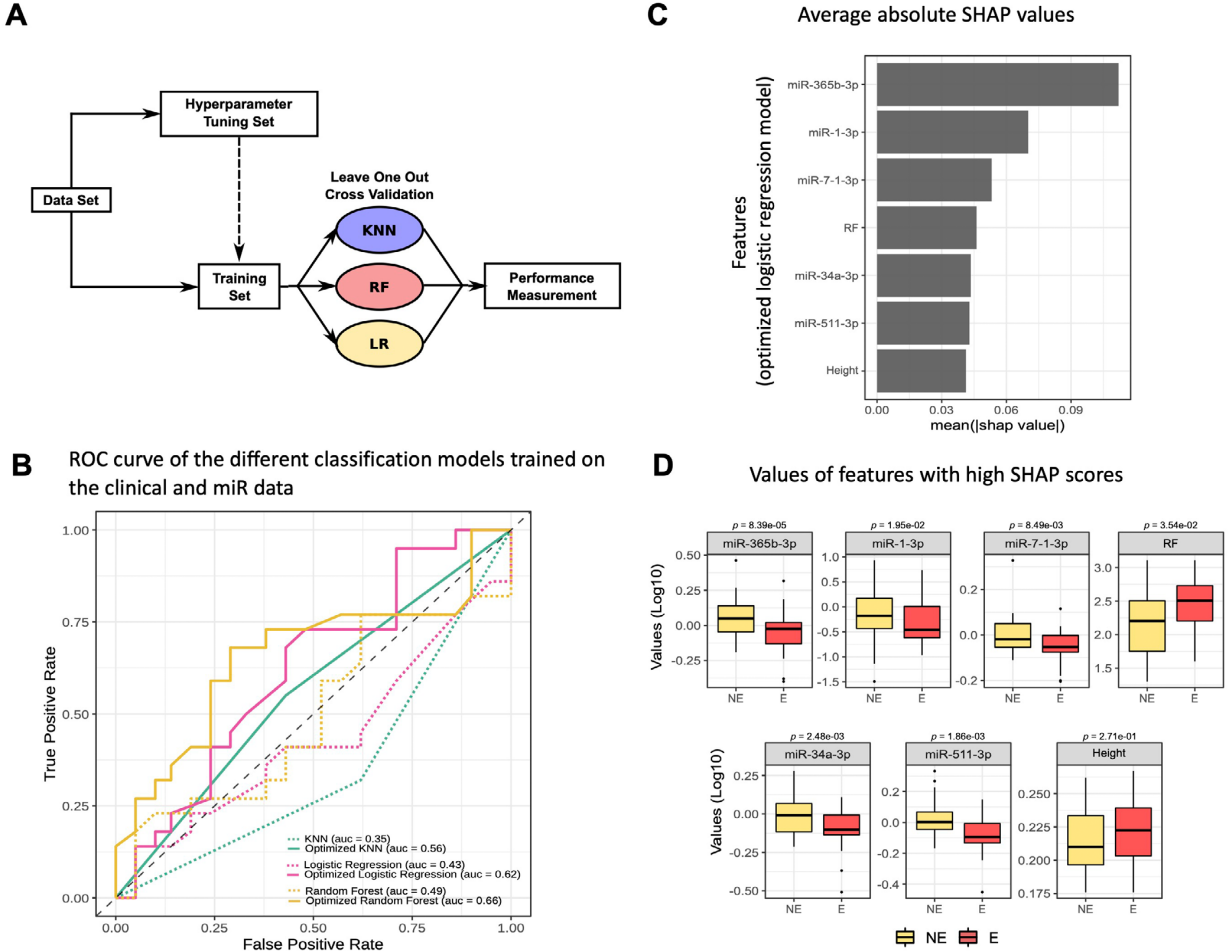
Machine‐learning to model a predictive tool for the development of bone erosions in rheumatoid arthritis (RA). (*A*) Machine learning: methods. Schematic describing the pipeline to build the classifiers. The model names are KNN for K nearest neighbor; RF for random forest; LR for logistic regression. (*B*) Receiver operating characteristic (ROC) curves of the different classification models trained on the clinical and microRNA (miR) data. The gray color represents the type of machine‐learning model (KNN, logistic regression, and random forest); the line type indicates whether the model was optimized or not. (*C*) Average absolute SHAP values related to the variables used to train the optimized logistic regression model. These SHAP values were computed using the SHAP python library. The variables were ordered in descending order of importance based on their SHAP values. (*D*) Distributions of values of features with high SHAP scores for the optimized logistic regression model (NE = nonerosive RA; E = erosive RA). The results (Log10 values) are presented as boxplots (median, min–max) (replication cohort, *n* = 86).

To investigate the predictive capacity of clinical features and of the 25 miRs selected from RNA‐Seq on the bone phenotype of RA, we generated classification models (logistic regression, KNN, and random forest), building three models trained on the raw data and three optimized models. These optimized models were generated using contributing variables identified by the feature selection step. The methodology is summarized in Figure 6*A*. By assessing the performance of these models, as determined by leave‐one‐out cross‐validation, we observed an expected increase in the classification performance of the optimized models (trained on the variables identified by feature selection) compared with the models trained on the raw data (from AUC = 0.35, AUC = 0.43, and AUC = 0.49 to AUC = 0.56, AUC = 0.62, and AUC = 0.66 for KNN, logistic regression and random forest models, respectively) (Fig. 6*B*). We thus selected the optimized random forest model because it presented the best performance (accuracy = 69.8%; sensitivity = 68.2%; specificity = 71.4%).

For the performance calculation, we used an optimal decision threshold of 0.49 calculated by the Youden's J statistic test that maximizes the specificity and sensitivity. We then computed SHAP values to interpret the classification decisions.^(^ ^31^ ^)^ These SHAP values reflect a measure of the feature importance in the model: a higher absolute value shows a higher contribution of the feature in the classification. The analysis showed that the selected features included, in decreasing order of importance, expression levels of miR‐365b‐3p, miR‐1‐3p, miR‐7‐1‐3p, RF, miR‐34a‐3p, and miR‐511‐3p (Fig. 6*C*). Rheumatoid factor titer ranked in the fourth position and was significantly higher in the erosive patients. Five of 7 of these features were expression levels of miRs found to be differentially expressed between erosive and nonerosive RA groups (Fig. 6*D*). When we trained a random forest model on the clinical data only, with a feature selection step omitting all 25 miRNA features, compared with our previous model, we observed a decrease in performance for this model (AUC = 0.52 versus AUC = 0.66).

**Fig. 7:**
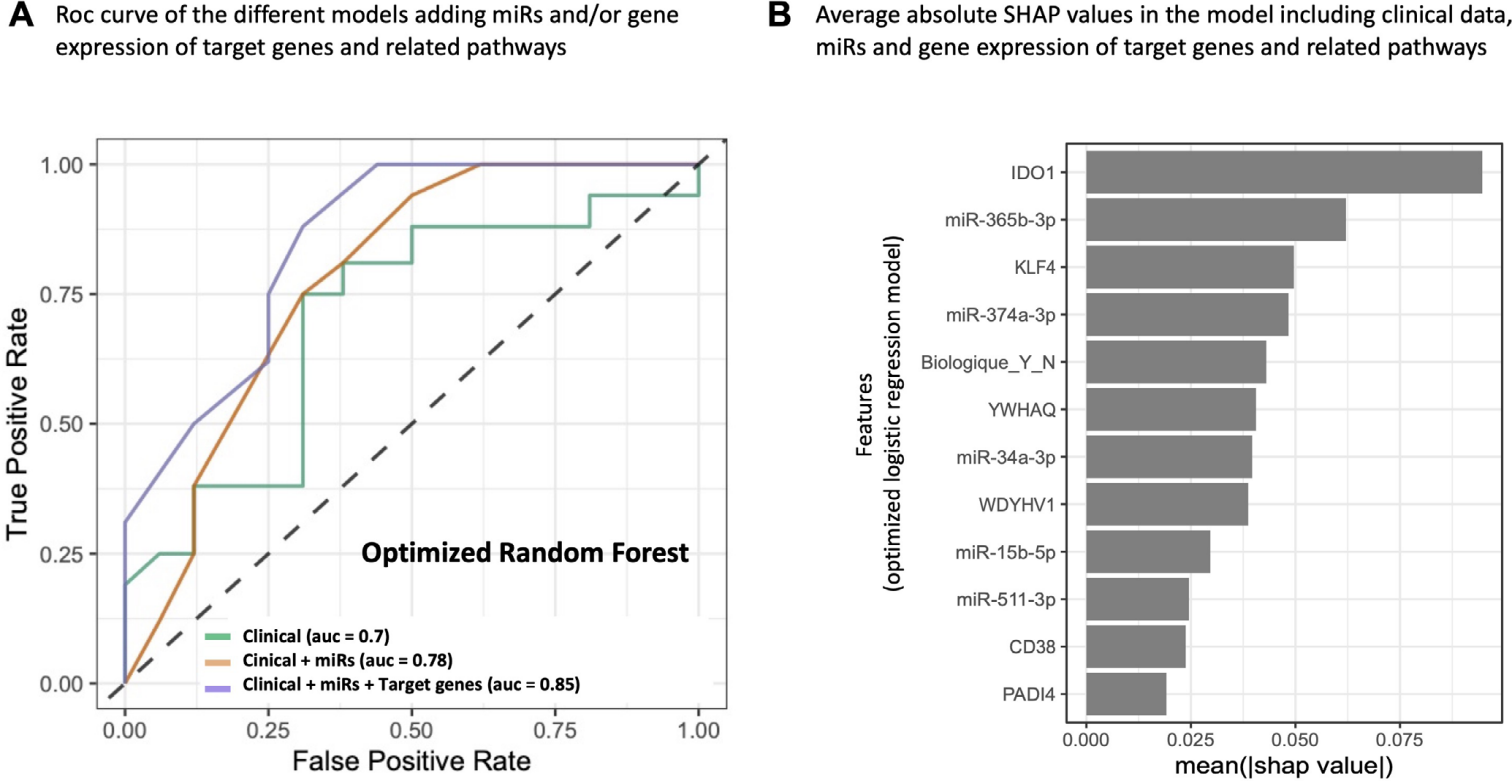
Receiver operating characteristic (ROC) curves of the different classification models considering or not gene expression. (*A*) A comparison of the models trained on the clinical data alone or with microRNA (miR) data, as well as with gene expression data from target genes and related pathways. For this model, the number of available samples was smaller (replication cohort, *n* = 60 patients for whom clinical data and expression of miRs and related genes were available). (*B*) Average absolute SHAP values related to the variables used to train the optimized logistic regression model. These SHAP values were computed using the SHAP python library. The variables were ordered in descending order of importance based on their SHAP values.

Finally, we also trained a random forest model including clinical data, miRs data, as well as gene expression data from target genes and related pathways. The limitation of this model is the smaller number of patients for whom all these data were available (60 patients versus 86 in the previous models). Using these 60 patients, the AUC values of the models were higher: using clinical data only (AUC = 0.70) and adding miRs (AUC = 0.78), with a further increased predictive value provided by adding the analysis of target genes and related pathways (AUC = 0.85) (Fig. 7*A*).

The expression of miR‐365b‐3p appears most influential in the miR‐based prediction model or in the 33‐gene association model. Potential candidate genes for osteoclast‐immune cell interactions, such as *IDO1* (indoleamine 2,3‐dioxygenase),^(^ ^45^ ^)^ KLF4 (KLF Transcription Factor 4),^(^ ^46^ ^)^ and PADI4 (Peptidyl Arginine Deiminase 4),^(^ ^47^ ^)^ appear to contribute to the predictive value of the model. Other genes also contributed, such as *WDYHV1* involved in N‐end rule proteolysis^(^ ^48^ ^)^ or YWHAQ encoding 14.3.3ζ^(^ ^49^ ^)^ (Fig. 7*B*). In summary, the expression levels of several miRs were found to be important contributing variables in our models of erosion prediction, complementing clinical information. The impact of miRs was increased by considering the expression of several of their target genes and related pathways.

## Discussion

Although they clearly associate with the development of bone erosions at a population level, the presence of specific antibodies, persistent inflammation, and intensity of treatment fail to predict erosive behavior in individual patients.^(^ ^5^, ^6^ ^)^ Our aim was to develop a personalized osteoclast‐related signature of erosive outcomes. To minimize the influence of known predictors, we compared miR expression profiles of PBMC‐derived osteoclasts from controls and from uniformly seropositive age‐ and sex‐matched RA patients with minimal disease activity but with extreme outcomes: either rapidly erosive or chronically nonerosive. In this proof‐of‐concept study, a genomewide analysis of miR expression followed by qPCR validation of 25 promising miRs, we identified decreases in miR‐34a‐3p, ‐365b‐3p, ‐374a‐3p and ‐511‐3p, and an increase in miR‐193b‐3p in osteoclasts from erosive RA patients compared with nonerosive RA patients or controls. Although none of these miRs are specific to osteoclasts, the tissue or cell specificity in which the miRs were analyzed, as well as the combined variation of several miRs, might be.^(^ ^3^ ^)^ The identification of a set of miRs characterizing a signature in the cells responsible for erosive lesions in arthritis should be highly relevant in characterizing the intrinsic properties of osteoclasts from subjects with erosive RA.

An integrative network analysis of the 5 miRs and their mRNA targets highlighted common pathways that were often closely or remotely related to the mTOR pathway. Indeed, although mTOR signaling is crucial in osteoclast differentiation and survival,^(^ ^50^ ^)^ TSC/mTORC1 also has a functional role in bone resorption, and osteolysis is prevented by administration of rapamycin, a potent inducer of autophagy via mTORC1 inhibition.^(^ ^51^ ^)^ Network analysis also identified genes related to potential osteoclast‐immune cell interactions, such as the ICOS‐ICOSL pathway,^(^ ^52^ ^)^ and genes involved in fusion and fission processes.^(^ ^43^, ^44^ ^)^

Based on this network analysis, we further complemented the phenotype of osteoclasts by analyzing the expression of the target genes of the miRs of interest, as well as certain genes associated with related pathways, such as mTOR signaling and immune cell–osteoclast interplay as mentioned above.

By integrating clinical and miR expression data of subjects with RA and controls, a machine‐learning approach using regression models brought out the predictive value of expected parameters such as RF titers but also highlighted the predominant value of some miRs (365b‐3p, 34a‐3p, and 511‐3p). The machine‐learning model trained on the clinical and miR expression data showed an accuracy value of 70% versus 55.8% for the model only trained on clinical data. The miR‐based model refined with the addition of genes directly targeted or belonging to common pathways modulated by the candidate miRs increased its accuracy up to 78%, with the reserve of the smaller number of patients compared with the other two models. Altogether, these findings suggest that miR expression and that of targets or related pathways may be additional relevant factors for a better prediction of the potential RA erosiveness. Although the accuracy of these models is not yet optimal, there is no better prediction model (AUC 0.63 to predict the risk of rapid progression in early RA^(^ ^6^ ^)^), and the evidence provided shows that modeling is possible and promising, using the intrinsic properties of osteoclasts.

This predictive model is robust and based on a rigorous logistic regression approach. It is a proof‐of‐concept that PBMC‐derived osteoclasts are different in patients with erosive versus nonerosive RA.

The expression of miR‐365b‐3p appears most influential in the miR‐based prediction model or in the 33‐gene association model. The expression of miR‐365b‐3p was found to correlate with that of miR‐374a‐3p, ‐511‐3p, and ‐34a‐3p, and showed a positive correlation with *MITF* and a negative correlation with *CD38, CD80*, and *SIRT1* genes. The positive correlation between *MITF* expression and several miRs was unexpected, as miRs contribute mainly to gene expression regulation by mediating gene silencing^(^ ^1^ ^)^ and because it suggests that there is a decrease in *MITF* expression in erosion‐prone osteoclasts. Indeed, the transcription factor MITF is essential to osteoclast development and maturation^(^ ^53^ ^)^ and also known to bind to the promoter of *DC‐STAMP*, a major transmembrane protein involved in osteoclast fusion and multinucleation.^(^ ^54^ ^)^ Although further mechanistic study is needed, one explanation for reconciling these data may involve factors known to repress *MITF* expression and to stimulate osteoclast formation as well, such as ATF4.^(^ ^55^, ^56^ ^)^ The expression profiles also suggest increases in *CD38* expression in mature osteoclasts of erosive RA. Previous studies in humans reported that *CD38* expression was induced during osteoclast differentiation. The use of anti‐CD38 for antimyeloma activity also reduced osteoclast formation and bone resorption in vitro.^(^ ^45^, ^57^ ^)^ *CD38* and *MITF* are not classical targets of the miRs identified here, justifying further study.

Genes of interest whose expression may contribute to the prediction of the erosive phenotype also include genes encoding proteins involved in the interaction between immune cells and osteoclasts: IDO1, which catalyzes tryptophan dioxygenation dampening the T‐cell response;^(^ ^45^ ^)^ PADI4, an enzyme involved in citrullination, contributing to ACPA‐induced osteoclast activation by binding directly to their citrullinated epitopes;^(^ ^47^ ^)^ and KLF4, a transcription factor that has been associated with osteoclast differentiation from dendritic cells.^(^ ^46^ ^)^

Among the candidate miRs, our results support the interest of miR‐374a‐3p with a strong decrease in its expression in osteoclasts from patients with erosive RA. The involvement of miR‐374 family members has been mainly described in tumorigenesis, including carcinoma of the digestive system.^(^ ^58^ ^)^ Few targets of miR‐374a‐3p have been validated, including *WNT3* in colon adenocarcinoma;^(^ ^59^ ^)^ *RUNX2*, a regulator of osteogenesis differentiation;^(^ ^60^ ^)^ and *AXIN2*, whose decreased expression might promote osteosarcoma.^(^ ^61^ ^)^

Finally, the potential interest of miR‐34a‐3p is also highlighted. Expression of miR‐34a‐3p has previously been associated with aging and downregulates *CD274*, encoding the immune checkpoint inhibitor PD‐L1,^(^ ^62^ ^)^ and *SIRT1*, encoding Sirtuin1, which is known to impede T_H_17^(^ ^63^ ^)^ and promote Foxp3 functions.^(^ ^64^ ^)^ Our in vitro analysis allowed us to confirm the direct impact of miR‐34a‐3p on the formation and resorption of human osteoclasts, as demonstrated in mice.^(^ ^35^ ^)^ The lack of significant impact of inhibiting the in vitro expression of the other miRs of interest was potentially due to indirect effects in a network of interactions, as considered in the integrative analysis and modeling.

The limitations of the study include several conceptual aspects. First, in the model we developed, the known predictive values of positive antibodies and CRP are attenuated, as most subjects included were in clinical remission, and all were seropositive. This likely contributed to the identification of a minor predictive value of RF titers and may also have amplified the role of miRs. An analysis in a larger unselected RA population would allow a more precise evaluation of the added value of miRs relative to the presence of antibodies and inflammation. Second, our study was transversal. Although bone resorption was not analyzed here in each patient, our study aimed to correlate osteoclast miRs to the bone phenotype in vivo, as biomarkers of erosions, referring to the endophenotype regardless of the behavior in vitro. Determination of the stability of miRs expression in in vitro osteoclasts from the same patient under various conditions of inflammation and treatment will need a longitudinal study. Third, the small sample size used in our analysis could affect the robustness of our model and explain some of the inconsistencies observed between the assessment of miRs by either RNA‐Seq or qPCR. Fourth, another limitation comes from the bulk RNA‐Seq analysis using the entire osteoclast culture, with cells that nevertheless belong to the monocyte–macrophage lineage and cultured in the presence of RANKL and MCSF, but heterogeneous with the presence of mono‐ and multinucleated cells, the advantage being that we study human cells. Finally, a predictive model that includes miRs and, moreover, the expression of their target genes from PBMC‐derived osteoclasts is not applicable in clinical practice. Our results must be viewed as a proof‐of‐concept that epigenetic modulation of osteoclasts may help personalize outcome prediction in RA patients. Although osteoclast miR signature could depend on upstream factors, epigenetic and genetic variations, our results provide evidence that the phenotype of PBMC‐derived osteoclasts obtained in vitro can differentiate erosive and nonerosive RA and that modeling is possible and promising, using the intrinsic properties of these cells. More easily accessible surrogates, in serum or PBMCs, will be needed to represent the profiles of osteoclasts associated with erosions, and our results will guide and facilitate the search for such surrogates.

In conclusion, we identified an osteoclast‐related miR signature associated with bone erosions in RA, which include the expression of some miRs, mainly miR‐365b‐3p, and that of genes involved in the mTOR pathway or in interactions with immune cells. Our results are highly relevant, as the implicated miRs and genes are expressed in osteoclasts, the culprits of bone erosions. Osteoclasts were generated outside the rheumatoid environment in long‐term culture; thus, our observations describe the intrinsic endophenotypes associated with erosion‐prone osteoclasts in RA. This proof‐of‐concept study indicates that RA subjects at risk of erosions may be better identified, suggesting a novel approach toward personalized treatment.

## Author Contributions

**Nguyen Hoang Dong:** Data curation; formal analysis; investigation; methodology; software; writing – original draft; writing – review and editing. **Lortie Audrey:** Data curation; formal analysis; investigation; resources; writing – review and editing. **Mbous Nguimbus Leopold:** Formal analysis; investigation; writing – review and editing. **Marrugo Javier:** Formal analysis; investigation; resources; writing – review and editing. **Allard‐Chamard Hugues:** Conceptualization; investigation; methodology; supervision; writing – review and editing. **Bouchard Luigi:** Conceptualization; formal analysis; methodology; supervision; writing – review and editing. **Boire Gilles:** Conceptualization; investigation; methodology; supervision; validation; writing – review and editing. **Michelle S Scott:** Conceptualization; formal analysis; funding acquisition; methodology; resources; software; supervision; writing – review and editing. **Roux Sophie:** Conceptualization; formal analysis; funding acquisition; investigation; methodology; resources; supervision; writing – original draft; writing – review and editing.

## Disclosures

The authors declare that they have no competing financial interests or personal relationships that could have appeared to influence the work reported in this article.

### Peer Review

The peer review history for this article is available at https://www.webofscience.com/api/gateway/wos/peer-review/10.1002/jbm4.10776.

## Supporting information


**Supplemental Table 1.** List of the 25 miRs Studied by qPCR.

**Supplemental Table 2.** List of Primers (Target Gene Expression).

**Supplemental Table 3.** Clinical and Demographic Variables Integrated in Machine Learning Models.
