# Correction to Osteoclast microRNA Profiling in Rheumatoid Arthritis to Capture the Erosive Factor

**DOI:** 10.1002/jbm4.10845

**Published:** 2023-12-01

Nguyen HD, Lortie A, Mbous Nguimbus L, et al. Osteoclast microRNA profiling in rheumatoid arthritis to capture the erosive factor. *JBMR Plus*. 2023;7:e10776. https://doi.org/10.1002/jbm4.10776

In the originally published version of the article, the author's names were inadvertently transposed. The correct author list appears below. The online version of this article has been corrected accordingly.

We apologize for this error.

Hoang Dong Nguyen,^1^ Audrey Lortie,^2^ Léopold Mbous Nguimbus,^2^ Javier Marrugo,^2^ Hugues Allard‐Chamard,^2^ Luigi Bouchard,^1^,^3^ Gilles Boire,^2^ Michelle S Scott,^1^ and Sophie Roux ^2^.

^1^Department of Biochemistry and Functional Genomics, University of Sherbrooke and Research Centre of the Centre Intégré Universitaire de Santé et Services Sociaux de l'Estrie – Centre Hospitalier Universitaire de Sherbrooke (CIUSSSE‐CHUS), Sherbrooke, Canada.

^2^Division of Rheumatology, Department of Medicine, Faculty of Medicine and Health Sciences, University of Sherbrooke and Research Centre of the Centre Intégré Universitaire de Santé et Services Sociaux de l'Estrie – Centre Hospitalier Universitaire de Sherbrooke (CIUSSSE‐CHUS), Sherbrooke, Canada.

^3^Department of Medical Biology, CIUSS du Saguenay‐Lac‐Saint‐Jean Hôpital Universitaire de Chicoutimi, Saguenay, Canada.

