# Supplementary material for: Osteoclast microRNA Profiling in Rheumatoid Arthritis to Capture the Erosive Factor
Source: JBMR Plus. 2023 Jun 5;7(8):e10776. doi: 10.1002/jbm4.10776 (PMC10443079; doi:10.1002/jbm4.10776)
Supplement: Supplementary file 1 — Supplemental Table 1. List of the 25 miRs Studied by qPCR. [file JBM4-7-e10776-s002.pdf]

Supplemental Table 1: List of the 25 miRs studied by qPCR

miR	Forward Sequence	Reverse Sequence
hsa-miR-106a-5p.SpFwd2	CGCTGCGTAAAAGTGCTTACAG[illegible]	GTGCAGGGTCCGAGGT
hsa-miR-1246.SpFwd1	GCTGTTCGAATGGATTTTTGG	GTGCAGGGTCCGAGGT
hsa-miR-152-3p.SpFwd2	GCTGTCGTCAGTGCATGACA	GTGCAGGGTCCGAGGT
hsa-miR-15b-5p.SpFwd1	GCGTAGCAGCACATCAT	GTGCAGGGTCCGAGGT
hsa-miR-193b-3p.SpFwd2	GCGCAACTGGCCCTCAAA	GTGCAGGGTCCGAGGT
hsa-miR-194-5p.SpFwd1	CGCTGCGTTGTAACAGCAACTC	GTGCAGGGTCCGAGGT
hsa-miR-200c-3p.SpFwd2	TGGTCGTAATACTGCCGGGTA	GTGCAGGGTCCGAGGT
hsa-miR-23ab-3p.SpFwd1	GCGCATCACATTGCCAGGG	GTGCAGGGTCCGAGGT
hsa-miR-15b-3p.SpFwd1	CGCTGCGTCGAATCATTATTTG	GTGCAGGGTCCGAGGT
hsa-miR-147b-5p.SpFwd1	CGCTGCGTTGGAAACATTTCTG	GTGCAGGGTCCGAGGT
hsa-miR-25-3p.SpFwd2	GCTGTCGCATTGCACTTGTCT	GTGCAGGGTCCGAGGT
hsa-miR-29b-3p.SpFwd3	GCGTAGCACCATTTGAAA	GTGCAGGGTCCGAGGT
hsa-miR-34a-3p.SpFwd1	CGCTGCGTCAATCAGCAAGTAT	GTGCAGGGTCCGAGGT
hsa-miR-365b-3p.SpFwd1	CGCTGCGTTAATGCCCCTAAAA	GTGCAGGGTCCGAGGT
hsa-miR-374a-5p.SpFwd2	GCGGCGTTTATAATACAACCTGA[illegible]	GTGCAGGGTCCGAGGT
hsa-miR-142-5p.SpFwd2	GCGGCGCATAAAGTAGAAAGC	GTGCAGGGTCCGAGGT
hsa-miR-454-3p.SpFwd1	CGCTGCGTTAGTGCAATATTG	GTGCAGGGTCCGAGGT
hsa-miR-511-3p.SpFwd1	CGCTGCGTAATGTGTAGCAA	GTGCAGGGTCCGAGGT
hsa-miR-7-1-3p.SpFwd1	TGGTCGGCAACAAATCACAGT	GTGCAGGGTCCGAGGT
hsa-miR-7706.SpFwd1	GCGTGAAGCGCCTGTGCTC	GTGCAGGGTCCGAGGT
hsa-miR-1-3p.SpFwd1	CGCTGCGTTGGAATGTAAAGAA	GTGCAGGGTCCGAGGT
hsa-miR-206.SpFwd1	CGCTGCGTTGGAATGTAAGGAA	GTGCAGGGTCCGAGGT
hsa-miR-374c-5p.SpFwd1	CGCTGCGTATAATACAACCTGC	GTGCAGGGTCCGAGGT
hsa-miR-374a-3p.SpFwd2	CGCTGCGTCTTATCAGATTGTA	GTGCAGGGTCCGAGGT
hsa-miR-17-5p.SpFwd2	GCTGTCGCAAAGTGCTTACAGT	GTGCAGGGTCCGAGGT
housekeeping gene		
small nuclear RNA U6 (snU6)	GCTCGCTTCGGCAGCACATA	ACGCTTCACGAATTTGCGTGTC
