# Supplementary material for: Osteoclast microRNA Profiling in Rheumatoid Arthritis to Capture the Erosive Factor
Source: JBMR Plus. 2023 Jun 5;7(8):e10776. doi: 10.1002/jbm4.10776 (PMC10443079; doi:10.1002/jbm4.10776)
Supplement: Supplementary file 2 — Supplemental Table 2. List of Primers (Target Gene Expression). [file JBM4-7-e10776-s003.pdf]

Supplemental Table 2: List of primers (target gene expression)

Supplemental Table 2: list of primers (target gene expression)

**genes**	protein / enzyme	**Forward Sequence**	**Reverse Sequence**
ABI2	Abl Interactor 2; actin cytoskeleton dynamics	TGCTGTGGTTGAGTATAGTGATCCTT	GGACAGCTCATCTTCCTTGTCTTTTG
ADA	Adenosine Deaminase	CGCTCACCCTTCCAGACTTCCT	CGCACCTCCACATACACCACG
AKT1	AKT Serine/Threonine Kinase 1	AGGGTTGGCTGCACAAACGAG	GCCACAGAGAAGTTGTTGAGGGG
AKT3	AKT Serine/Threonine Kinase 3	TTGGTGGAGGACCAGATGATGC	ATCTTGCCAGTTTACTCCAGAGAAG
ANKRD11	Ankryin repeat domain-containing protein	CGAGCCGCCATCCGCG	CGTCGTAGTAGCCCCGGTTACA
AXIN2	Axin-related protein,beta-catenin stability	GCCACACCCTTCTCCAATCCAA	AGCCTTCAGCATCCTCCGGT
CCND1	Cyclin D1; regulator of CDK kinases	CCTCACACGCTTCCTCTCCAGA	CTCCTCCTCTTCCTCCTCCTCG
CD200	CD200	AGAATCAGGTGGGGAAGGAGGT	TTGAGATTAGGACGAGAAGAATTACCA
CD274	CD274	AGGCTCCGCACCAGCCG	CGTTCAGCAAATGCCAGTAGGT
CD38	CD38	TCCGGGCACCACCAAGCG	ATGCACCCTTGAAAGCATCCCA
CD80	CD80	CGCAGAAGCAAGGGGCTGAAAA	ACGTAAAGGGCAAGGTGGGGT
CD86	CD86	ACCAACACAATGGAGAGGGAAGAGA	TCGAACTTTTAAAAACACGCTGGGC
CSF1R	MCSF-R	CCCCCAGCCCAACGTGACAT	CAGGCTCTGCACCGTCACCT
DOK2	Docking Protein 2	GGCGCTTTGGGCGGGACAAG	GCAGAGATGGCCTCTTCCAGGG
ENTPD1	Ectonucleoside Triphosphate Diphosphohydrolase 1	GCAACTACCCCTTTGACTTCCA	GGACTATGCTGAACCACCTTGT
GXYLT1	Xylosyltransferase	TGCTGAAGATCAGCTACATCATAGCT	TTCTCACTTGGAAAGGTTATGGGGT
ICOSLG	Inducible T Cell Costimulator Ligand	GGGCTTGTATGACGTGGTCAGC	TGTGATCTTGTCTCTCTCTCCGA
IDO1	Indoleamine 2,3-Dioxygenase 1	AAGGAGTTTGCAGGGGGCAGT	AAGTTCCTGTGAGCTGGTGGCA
KLF4	Kruppel Like Factor 4	CCCATCTCAAGGCACACCTGC	CATTTTTGGCACTGGAACGGGC
MITF	Melanocyte Inducing Transcription Factor	CGCCAACCGGCATTTGTTGC	AATCTGGAGAGCAGAGACCCGT
NT5E	5'-Nucleotidase Ecto	TTGCACTGGGACATTCGGGTTT	GGCACCTCTTTGGAAGGTGGATT
PADI4	Peptidyl Arginine Deiminase 4	GCCAAGAAGAAATCCACAGGTTC	TGGTGGAGTCTTGGGTCCGTAG
PLEKHA2	Pleckstrin Homology Domain Containing A2	AAGATATGAAGGACTGGGTTGAAGCC	GAACTTCAGTGGTCATGGGTAGGC
PRKAA1	Protein Kinase AMP-Activated Catalytic Subunit Alpha	GTGTACGAAGGAAGAATCCTGTGACA	GGAGTAGCAGTCCCTGATTTGGC
PRKAA2	Protein Kinase AMP-Activated Catalytic Subunit Alpha	TTGACCCACTGAAACGAGCAACT	TTCACACACTTCTTTCACAGCCTCA
PTEN	Phosphatase And Tensin Homolog	ATTCCCAGTCAGAGGCGCTAT	TCATCTTGTGAAACAACAGTGCCA
SHCBP1	SHC Binding And Spindle Associated 1	AGGAGCTGTGGGTGGTGTTTGA	TCATCCTCCTCTTCTTCATCCCAACT
SIRT1	Sirtuin1	GCTGGAACAGGTTGCGGGAATC	TCTCCTCGTACAGCTTCACAGTCA
SNRK	sucrose nonfermenting-related kinase	TCATTCAGCGCATGGTGCTTGG	CAGCAGGAAGTATGTGGCTGTGA
SOX5	SRY-Box Transcription Factor 5	AACAGGCGGCAGGAAATGCG	GGCAGGTGAGGGGAGGGCAT
TNFRSF14	TNF Receptor Superfamily Member 14	AGCTGCCGGTCTGAGCCTGA	CTGGGGCGTAGCAGGGGGC
WDYHV1	NTAQ1, N-Terminal Glutamine Amidase 1	GTCTGATGATGACATTCACCCACAGT	CAATGCAGGGATATGGCGGCG
YWHAQ	Tyrosine 3-Monooxygenase/Tryptophan 5-Monooxygenase Activation Protein Theta	AAAGTGGAGTCCGAGCTGAGAT	ACTCTCTGGATTAGTTGCATTGGC
housekeeping genes			
MRPL19	Mitochondrial Ribosomal Protein L19	AAGGAGAAAAGTACTCCACATTCCAGAG	TGGGTCAGCTGTAGTAACACGA
YWHAZ	Tyrosine 3-Monooxygenase/Tryptophan 5-Monooxygenase Activation Protein Zeta	TCCCCAATGCTTCACAAGCAGA	TCTTGTCATCACCAGCGGCAA
PUM1	Pumilio homolog 1	TGAGGTGTGCACCATGAAC	CAGAATGTGCTTGCCATAGG

In the shaded boxes are the selected genes concomitantly regulated by the 5 miRs whose expression levels were different between groups (E, NE, controls).
