# Supplementary material for: Osteoclast microRNA Profiling in Rheumatoid Arthritis to Capture the Erosive Factor
Source: JBMR Plus. 2023 Jun 5;7(8):e10776. doi: 10.1002/jbm4.10776 (PMC10443079; doi:10.1002/jbm4.10776)
Supplement: Supplementary file 3 — Supplemental Table 3. Clinical and Demographic Variables Integrated in Machine Learning Models. [file JBM4-7-e10776-s001.pdf]

Supplemental Table 3: Clinical and demographic variables integrated in machine learning models

Supplementary Table 3: Clinical and demographic variables integrated in machine learning models

Sex	RF
Age	Anti-CCP
Weight (kg)	CRP
Height (m)	Methotrexate use
BMI	Methotrexate dose
Menopausal status	DMARD (other) use
Smoking (no, active, past)	DMARD name
Alcohol (no, active, past)	Biologics use
Serum 25OH-VitD	Biologics name
Morning stiffness	Prednisone use
SJC66	Prednisone dose
TJC68	regular NSAID use
HAQ	Osteoporosis treatment (Biphosphonate, denosumab) use
DAS28	Osteoporosis treatment (Biphosphonate, denosumab) name
	Calcium-vitamin D use
